# Some Results on Cumulative Residual Inaccuracy Measure of *k*-Record Values

**DOI:** 10.3390/e28010017

**Published:** 2025-12-24

**Authors:** Ritu Goel, Vikas Kumar, Sarang Vehale, Tony C. Scott

**Affiliations:** 1 School of Engineering and Technology, Vivekananda Institute of Professional Studies-Technical Campus, Pitampura, Delhi 110034, India; ritujindalmahe@gmail.com; 2 Department of Applied Sciences, UIET, M.D. University, Rohtak 124001, India; vikas_iitr82@yahoo.co.in; 3 School of Cyber Security & Digital Forensics, National Forensic Sciences University, Delhi 110085, India; sarangvehale2@gmail.com; 4 Institut für Physikalische Chemie, RWTH-Aachen University, 52056 Aachen, Germany

**Keywords:** cumulative residual entropy, inaccuracy measure, k-record value, hazard rate

## Abstract

Herein, we consider the significance of cumulative residual entropy (CRE) and its numerous generalizations. This article presents an extension of the cumulative residual inaccuracy to *k*-record values. We examine certain properties of this measure. Additionally, we investigate some stochastic ordering and identify the proposed measure for several distributions that frequently arise in various realistic scenarios and have applications across multiple fields of science and engineering.

## 1. Introduction

The pivotal event that established the field of information theory was the 1948 seminal paper by Claude E. Shannon [1], in which he presented the concept of uncertainty for a continuous random variable *X* with a probability density function f(x) as follows:

(1) $$H\left(f\right)=-\int_{0}^{\infty }f\left(x\right)\ln f\left(x\right)dx\;.$$

After the work of Shannon, there was enormous development in this field, resulting in a huge literature introducing numerous information-theoretic measures by various researchers. Kerridge [2] provides the inaccuracy measure, which is a generalization of Shannon entropy, as follows:

(2) $$H\left(f,g\right)=-\int_{0}^{\infty }f(x)\ln g(x)dx.$$

Here, f(x) is the actual distribution, whereas g(x) is the predicted one. In order to address certain limitations associated with Shannon’s entropy measure, Rao et al. [3] introduced a novel measure of uncertainty, referred to as the cumulative residual entropy. In this measure, the probability density function f(x) has been substituted with the survival function (a function that gives the probability that a patient, device, or other object of interest will survive past a certain time) $\bar{F}\left(x\right)$ of the random variable *X*, defined as

(3) $$H\left(\bar{F}\right)=-\int_{0}^{\infty }\bar{F}\left(x\right)\ln\bar{F}\left(x\right)dx.$$

This measure of uncertainty is especially relevant for characterizing the information in issues related to the aging characteristics of reliability theory, which is based on the mean residual life function. Following Rao’s introduction of this cumulative residual entropy [3], it has garnered significant attention from researchers. Asadi and Zohrev [4] examined its dynamic variant to elucidate the effect of age on the information pertaining to the residual lifetime of a system or component. In a similar vein to cumulative residual entropy (3), Dicrescenzo and Longobardi [5] presented cumulative entropy as particularly applicable to problems associated with inactivity time. Sunoj and Linu [6] introduced the cumulative version of Renyi’s entropy. Taneja and Kumar [7] expanded the concept of cumulative residual entropy to include the cumulative residual inaccuracy (CRI) and, subsequently, dynamic cumulative inaccuracy (DCI), while also investigating some of its properties. The measure proposed by Taneja and Kumar [7] is defined as follows:

(4) $$H\left(\bar{F},\bar{G}\right)=-\int_{0}^{\infty }\bar{F}\left(x\right)\ln\bar{G}\left(x\right)dx\;.$$

Consider a sequence of independent and identically distributed continuous random variables denoted as $\left\{X_{i},\;i\geq 1\right\}$, with a distribution function F(x) and a probability density function f(x). An observation $X_{j}$ is referred to as an upper record value if its value exceeds that of all preceding observations. A similar definition applies to a lower record value. However, in various scenarios where the expected waiting time between two record values is significantly large, this model of record values becomes inadequate. To address such situations, a novel model of *k*-records has been introduced by Dziubdziela and Kopocinski [8]. They define the $n^{th}$ upper *k*-record as follows. For a positive integer *k*, define $T_{1,k}=k$, and for n≥2,

$$T_{n,k}=min\left\{j:j>T_{n-1,k};\;X_{j-k+1:j}>X_{T_{n-1,k}-k+1:T_{n-1,k}}\right\}\;.$$

Let $Y_{n,k}=X_{T_{n,k}-k+1:T_{n,k}},n\geq 1$, where $X_{i:n}$ denotes the $i^{th}$ order statistic in a sample of size *n*. The sequence $\left\{Y_{n,k},n\geq 1\right\}$ is referred to as *k*-record values. An analogous definition can be given for a lower *k*-record value. If we put k=1, the results for usual records can be obtained as a special case.

In the existing literature, several authors have studied cumulative inaccuracy measures for record values. For instance, Eskandarzadeh, Di Crescenzo, and Tahmasebi [9] introduced a cumulative measure of inaccuracy involving the distribution function, defined as follows:

$$I\left(F_{n,k},F\right)=-\int F_{n,k}\left(x\right)\ln F\left(x\right)\;dx,$$

where $F_{n,k}\left(x\right)$ and F(x) are the cumulative distribution functions of the *k*-th record value and the parent variable, respectively.

If we set k=2 or k=3 in the model of an upper *k*-record value, this means that instead of viewing the largest value, we observe the second or third largest values. We can take the values of *k* that are of great importance in the context of a particular problem. Claims in some non-life insurance can be used as an example (see Kamps [10]). So, the concept of *k*-record values has been widely studied in the literature (refer to Berred [11] and Fashandi and Ahmadi [12]). The probability distribution function (pdf) and the survival function of the $n^{th}$ upper *k*-record are given, respectively, as follows:

(5) $$f_{n,k}\left(x\right)=\frac{k^{n}}{\Gamma (n)}{\left(-\ln\bar{F}\left(x\right)\right)}^{n-1}{\left(\bar{F}\left(x\right)\right)}^{k-1}f\left(x\right)$$

and

(6) $${\bar{F}}_{n,k}=\sum_{j=0}^{n-1}\frac{1}{j!}{\left(\bar{F}\left(x\right)\right)}^{k}{\left(-k\ln\bar{F}\left(x\right)\right)}^{j}$$

where Γ is the (complete) Gamma function (e.g., see Arnold et al. [13]). Psarrakos and Navarro [14] expanded the notion of CRE by connecting it to the average duration between record values and the idea of the *relevation transform* [15,16]. The latter describes the total lifetime of a component replaced by a new one of the same age upon failure. Tahmasebi and Eskandarzadeh [17] suggested an enhancement of cumulative entropy based on the $k^{th}$ lower record values, while also examining its dynamic variant that incorporates the past lifetime. Goel et al. [18] investigated the measure of inaccuracy between the record distribution and the parent distribution, as well as between the *k*-record distribution and the parent distribution.

In this article, we study the cumulative residual inaccuracy contained in the sequence of *k*-record values. The structure of this paper is outlined as follows. In Section 2, we introduce the extension of the cumulative residual inaccuracy measure to *k*-record values. In Section 3, we examine several properties of the proposed measure and establish some bounds for it. Subsequently, in Section 4, we investigate certain aspects of stochastic ordering. Section 5 presents a simplified expression for cumulative residual inaccuracy to facilitate computations and calculations. Section 6 presents an application to extremal quantum uncertainty. Finally, conclusive comments are made at the end in Section 7.

## 2. Cumulative Residual Inaccuracy Measure

Corresponding to the measure (4), we introduce the cumulative residual inaccuracy measure between the *k*-record distribution ${\bar{F}}_{n,k}\left(x\right)$ and the parent distribution $\bar{F}$ as follows:

(7) $$H\left({\bar{F}}_{n,k},\bar{F}\right)=-\int_{0}^{\infty }{\bar{F}}_{n,k}\left(x\right)\ln\bar{F}\left(x\right)dx.$$

Using (6), we have

(8) $$\begin{array}{cc} H({\bar{F}}_{n,k},\bar{F}) & =-\int_{0}^{\infty }\sum_{j=0}^{n-1}\frac{1}{j!}{\left(\bar{F}\left(x\right)\right)}^{k}{\left(-k\ln\bar{F}\left(x\right)\right)}^{j}\ln\bar{F}\left(x\right)dx\\ & =\sum_{j=0}^{n-1}\int_{0}^{\infty }\frac{k^{j}}{j!}{\left(\bar{F}\left(x\right)\right)}^{k}{\left(-\ln\bar{F}\left(x\right)\right)}^{j+1}dx\;. \end{array}$$

Here and throughout we write $f\left(x\right)=F^{\prime }\left(x\right)$ for the density associated with the distribution function F(x) and

$$\bar{F}\left(x\right)=1-F\left(x\right)$$

for the survival (tail) function. The hazard (failure rate) function corresponding to *F* is defined by

$$\lambda_{F}\left(x\right)=\frac{f(x)}{\bar{F}\left(x\right)},\;x\geq 0,$$

so that $f\left(x\right)=\lambda_{F}\left(x\right)\;\bar{F}\left(x\right)$. It will be convenient to identify the quantity

$$f_{j+2,k}\left(x\right):=\frac{k^{j+2}}{\Gamma (j+2)}{\left(-\ln\bar{F}\left(x\right)\right)}^{j+1}{\left(\bar{F}\left(x\right)\right)}^{k}\lambda_{F}\left(x\right),$$

which (upon using $f\left(x\right)=\lambda_{F}\left(x\right)\bar{F}\left(x\right)$) equals

$$\frac{k^{j+2}{\left(-\ln\bar{F}\left(x\right)\right)}^{j+1}{\left(\bar{F}\left(x\right)\right)}^{k-1}f\left(x\right)}{\Gamma (j+2)}.$$

After some rearrangements, we get

(9) $$\begin{array}{cc} H({\bar{F}}_{n,k},\bar{F}) & =\sum_{j=0}^{n-1}\int_{0}^{\infty }\frac{\left(j+1\right)}{k^{2}\lambda_{F}\left(x\right)}.\frac{k^{j+2}{\left(-\ln\bar{F}\left(x\right)\right)}^{\left(j+1\right)}{\left(\bar{F}\left(x\right)\right)}^{\left(k-1\right)}f\left(x\right)}{\Gamma (j+2)}dx\\ \; & =\sum_{j=0}^{n-1}\int_{0}^{\infty }\frac{\left(j+1\right)}{k^{2}\lambda_{F}\left(x\right)}f_{j+2,k}\left(x\right)\;dx\\ \; & =\sum_{j=0}^{n-1}\frac{\left(j+1\right)}{k^{2}}E_{f_{j+2,k}}\left(\frac{1}{\lambda_{F}\left(x\right)}\right)\;. \end{array}$$

Here, *E* stands for expectation and $\lambda_{F}\left(x\right)$ stands for the *hazard function* corresponding to F(x). The hazard function (also known as the failure rate, hazard rate, or force of mortality) measures the likelihood of, for example, death or failure occurring at a specific time, given that the event has not occurred before that time [19,20].

## 3. Properties and the Bounds of the Measure

In the present section, we study some of the properties of the proposed measure of cumulative inaccuracy as follows:

1. $H\left({\bar{F}}_{n,k},\bar{F}\right)=\sum_{j=0}^{n-1}k^{j}\left(j+1\right)\left(\mu_{j+2,k}\left(x\right)-\mu_{j+1,k}\left(x\right)\right),$ where $\mu_{n,k}\left(x\right)=\int_{0}^{\infty }{\bar{F}}_{n,k}\left(x\right)dx.$
  **Proof**. From (6), we can write
  (10) $${\bar{F}}_{j+2,k}\left(x\right)-{\bar{F}}_{j+1,k}\left(x\right)={\left(\bar{F}\left(x\right)\right)}^{k}\frac{{\left(-k\ln\bar{F}\left(x\right)\right)}^{j+1}}{(j+1)!}.$$
  Therefore from (8) and (10), we get
  $$\begin{array}{cc} H({\bar{F}}_{n,k},\bar{F}) & =\sum_{j=0}^{n-1}k^{j}\left(j+1\right)\int_{0}^{\infty }\left({\bar{F}}_{j+2,k}\left(x\right)-{\bar{F}}_{j+1,k}\left(x\right)\right)dx\\ \; & =\sum_{j=0}^{n-1}k^{j}\left(j+1\right)\left(\mu_{j+2,k}\left(x\right)-\mu_{j+1,k}\left(x\right)\right)\;. \end{array}$$
  □
  **Remark** **1.** *If for a fixed k, $F_{n,k}$ is a decreasing function of n, that is, ${\bar{F}}_{n,k}$ is an increasing function of n, then ${\bar{F}}_{j+2,k}>{\bar{F}}_{j+1,k}$. From the previous result, we can see that $H({\bar{F}}_{n,k},\bar{F})$ is an increasing function of n.*
2. Consider two random variables *X* and *Y* with survival functions $\bar{F}\left(x\right)$ and $\bar{G}\left(y\right)$, respectively, such that Y=ϕ(X), where ϕ is a strictly increasing function and differentiable almost everywhere with ϕ(0)=0, then
  (11) $$H\left({\bar{G}}_{n,k},\bar{G}\right)\;=\;\sum_{j=0}^{n-1}\int_{0}^{\infty }\frac{k^{j}}{j!}{\left(\bar{F}\left(x\right)\right)}^{k}{\left(-\ln\bar{F}\left(x\right)\right)}^{j+1}\phi \prime \left(x\right)dx.$$
  **Proof**. From (8), we can write
  (12) $$H\left({\bar{G}}_{n,k},\bar{G}\right)\;=\;\sum_{j=0}^{n-1}\int_{0}^{\infty }\frac{k^{j}}{j!}{\left(\bar{G}\left(y\right)\right)}^{k}{\left(-\ln\bar{G}\left(y\right)\right)}^{j+1}dy\;.$$
  Now Y=ϕ(X) ⇒ $\bar{G}\left(y\right)=\bar{F}\left(x\right)$ and ${\bar{G}}_{n,k}\left(y\right)\;=\;{\bar{F}}_{n,k}\left(x\right)$. Also dy=ϕ′(x)dx.
  By setting all these values in (12), the result is obvious. □
  **Remark** **2.** *In particular, Y=ϕ(X)=aX ⇒ ϕ′(x)=a. Therefore (11) becomes*
  $$\begin{array}{cc} H({\bar{G}}_{n,k},\bar{G})\; & =\;\sum_{j=0}^{n-1}a\int_{0}^{\infty }\frac{k^{j}}{j!}{\left(\bar{F}\left(x\right)\right)}^{k}{\left(-\ln\bar{F}\left(x\right)\right)}^{j+1}dx\\ \; & =a\;H({\bar{F}}_{n,k},\bar{F}). \end{array}$$
3. If $\bar{G}\left(x\right)={\left(\bar{F}\left(x\right)\right)}^{\beta }$, where β is an integer greater than 1 and $\bar{F}\left(x\right)$ and $\bar{G}\left(x\right)$ are the survival functions of *X* and *Y*, respectively, then
  $$H\left({\bar{G}}_{n,k},\bar{G}\right)=\beta H\left({\bar{F}}_{n,k\beta },\bar{F}\right)$$
  **Proof**. We know that
  $$\begin{array}{cc} H({\bar{G}}_{n,k},\bar{G}) & \;=\;\sum_{j=0}^{n-1}\int_{0}^{\infty }\frac{k^{j}}{j!}{\left(\bar{G}\left(x\right)\right)}^{k}{\left(-\ln\bar{G}\left(x\right)\right)}^{j+1}dx\\ \; & =\sum_{j=0}^{n-1}\int_{0}^{\infty }\frac{k^{j}}{j!}{\left(\bar{F}\left(x\right)\right)}^{k\beta }{\left(-\beta \ln\bar{F}\left(x\right)\right)}^{j+1}dx\\ \; & =\beta \sum_{j=0}^{n-1}\int_{0}^{\infty }\frac{{\left(k\beta \right)}^{j}}{j!}{\left(\bar{F}\left(x\right)\right)}^{k\beta }{\left(-\ln\bar{F}\left(x\right)\right)}^{j+1}dx\\ \; & =\beta H({\bar{F}}_{n,k\beta },\bar{F})\;. \end{array}$$
  □
4. Consider
  (13) $$\eta \left(X\right)=-\int_{0}^{\infty }{\left(\bar{F}\left(x\right)\right)}^{k}\ln\bar{F}\left(x\right)\;dx,$$
  Then
  (14) $$H\left({\bar{F}}_{n,k},\bar{F}\right)\geq \sum_{j=0}^{n-1}\frac{k^{j}}{j!}\frac{{\left(\eta (X)\right)}^{j+1}}{C_{k}^{j}},\;\;Where\;\;C_{k}=\int_{0}^{\infty }{\left(\bar{F}\left(x\right)\right)}^{k}dx$$
  **Proof**. From (8),
  (15) $$H\left({\bar{F}}_{n,k},\bar{F}\right)=\sum_{j=0}^{n-1}\int_{0}^{\infty }\frac{k^{j}}{j!}{\left(\bar{F}\left(x\right)\right)}^{k}{\left(-\ln\bar{F}\left(x\right)\right)}^{\;j+1}dx.$$
  Since $0\leq \bar{F}\left(x\right)\leq 1$ for a survival function, we have $\bar{F}\left(x\right)\geq {\left(\bar{F}\left(x\right)\right)}^{j+1}$ for every integer j≥0. Thus,
  $$H\left({\bar{F}}_{n,k},\bar{F}\right)\geq \sum_{j=0}^{n-1}\int_{0}^{\infty }\frac{k^{j}}{j!}{\left(\bar{F}\left(x\right)\right)}^{k-1}{\left(\bar{F}\left(x\right)\right)}^{j+1}{\left(-\ln\bar{F}\left(x\right)\right)}^{\;j+1}dx.$$
  To apply Jensen’s inequality [21], define
  $$C_{k}=\int_{0}^{\infty }{\left(\bar{F}\left(x\right)\right)}^{k}dx>0,\;Z\left(x\right)=-\ln\bar{F}\left(x\right)\geq 0,$$
  and the probability measure
  $$dP_{k}\left(x\right)=\frac{{\left(\bar{F}\left(x\right)\right)}^{k}}{C_{k}}\;dx.$$
  Let $\phi \left(t\right)=t^{j+1}$, which is convex for all j≥0. Jensen’s inequality then gives
  $$\int_{0}^{\infty }Z{\left(x\right)}^{j+1}dP_{k}\left(x\right)\;\geq \;{\left(\int_{0}^{\infty }Z\left(x\right)dP_{k}\left(x\right)\right)}^{j+1}.$$
  Multiplying both sides by $C_{k}$ and using
  $$\eta \left(X\right)=\int_{0}^{\infty }{\left(\bar{F}\left(x\right)\right)}^{k}Z\left(x\right)dx,$$
  yields
  $$\int_{0}^{\infty }{\left(\bar{F}\left(x\right)\right)}^{k}{\left(-\ln\bar{F}\left(x\right)\right)}^{j+1}dx\;\geq \;\frac{\eta {\left(X\right)}^{\;j+1}}{C_{k}^{j}}.$$
  Substitute this bound into the expression (15).
  This completes the proof. □
5. Let *X* denote an absolutely continuous non-negative random variable; then
  (16) $$H\left({\bar{F}}_{n,1},\bar{F}\right)\geq \sum_{j=0}^{n-1}\frac{{\left(H(\bar{F})\right)}^{j+1}}{j!\;{\left(E(X)\right)}^{j^{\prime }}}$$
  where E(X) denotes the expectation of *X* and $H(\bar{F})$ is defined in (3).
  **Proof**. This result follows directly from (14) by taking k=1. For k=1, we have
  $$C_{1}=\int_{0}^{\infty }\bar{F}\left(x\right)\;dx=E\left(X\right),$$
  and
  $$\eta \left(X\right)=-\int_{0}^{\infty }\bar{F}\left(x\right)\ln\bar{F}\left(x\right)\;dx\;,$$
  which is the cumulative residual entropy. Hence,
  $$H\left(\bar{F}\right)=-\int_{0}^{\infty }\bar{F}\left(x\right)\ln\bar{F}\left(x\right)\;dx.$$
  Substituting these expressions into (14) completes the proof. □
6. Let *X* be an absolutely continuous non-negative random variable with
  E(X)=1.
  Then
  (17) $$H\left({\bar{F}}_{n,1},\bar{F}\right)\geq \sum_{j=0}^{n-1}\frac{{\left(H(\bar{F})\right)}^{j+1}}{j!},$$
  where $H(\bar{F})$ is given by (3).
  **Proof**. Setting k=1 in (14), we obtain
  $$C_{1}=\int_{0}^{\infty }\bar{F}\left(x\right)\;dx=E\left(X\right)=1\;.$$
  Moreover,
  $$\eta \left(X\right)=-\int_{0}^{\infty }\bar{F}\left(x\right)\ln\bar{F}\left(x\right)\;dx=H\left(\bar{F}\right),$$
  which is the cumulative residual entropy. Substituting these values into (14) yields the desired result. □

## 4. Some Results on Stochastic Ordering

In this section, we prove some order properties of the cumulative inaccuracy measure for *k*-record values. First we provide the following definitions.

**Definition** **1.** *A random variable X is said to be less than Y in the stochastic ordering denoted by $X\overset{st}{\leq }Y$ if $\bar{F}\left(x\right)\leq \bar{G}\left(x\right)$ for all x, where $\bar{F}\left(x\right)$ and $\bar{G}\left(x\right)$ are the survival functions of X and Y, respectively.*

**Definition** **2.** *A random variable X is said to be less than Y in the likelihood ratio ordering denoted by $X\overset{lr}{\leq }Y$ if $\frac{f_{X}\left(x\right)}{g_{Y}\left(x\right)}$ is non-increasing in x, where $f_{X}\left(x\right)$ and $g_{Y}\left(x\right)$ are the pdfs of X and Y, respectively.*

**Proposition** **1.** *If $E(X_{n,k})$ and E(X) denote the expected value of the $n^{th}$ k-record value and the parent distribution such that $X_{n,k}\overset{st}{\leq }X$, then*

(18) $$\left(i\right)\;H\left({\bar{F}}_{n,k}\right)\leq H\left({\bar{F}}_{n,k},\bar{F}\right)-E\left(X_{n,k}\right)\ln\frac{E(X_{n,k})}{E(X)}.$$

(19) $$\left(ii\right)\;H\left({\bar{F}}_{n,k}\right)\leq H\left(\bar{F}\right)-E\left(X_{n,k}\right)\ln\frac{E(X_{n,k})}{E(X)}.$$

*Here $H({\bar{F}}_{n,k})$ and $H(\bar{F})$ denote the cumulative residual entropy for the random variables $X_{n,k}$ and X, respectively.*

**Proof**. By the log-sum inequality, we have

$$\begin{array}{cc} \int_{0}^{\infty }{\bar{F}}_{n,k}\left(x\right)\ln\frac{{\bar{F}}_{n,k}\left(x\right)}{\bar{F}\left(x\right)}dx & \geq \int_{0}^{\infty }{\bar{F}}_{n,k}\left(x\right)dx\;\ln\frac{\int_{0}^{\infty }{\bar{F}}_{n,k}\left(x\right)dx}{\int_{0}^{\infty }\bar{F}\left(x\right)dx}\\ \; & =E\left(X_{n,k}\right)\ln\frac{E(X_{n,k})}{E(X)}. \end{array}$$

Hence, using the previous inequality, we obtain

$$\begin{array}{cc} H({\bar{F}}_{n,k}) & =-\int_{0}^{\infty }{\bar{F}}_{n,k}\left(x\right)\ln{\bar{F}}_{n,k}\left(x\right)\;dx\\ \; & \leq -\int_{0}^{\infty }{\bar{F}}_{n,k}\left(x\right)\ln\bar{F}\left(x\right)\;dx-E\left(X_{n,k}\right)\ln\frac{E(X_{n,k})}{E(X)}\\ \; & =H\left({\bar{F}}_{n,k},\bar{F}\right)-E\left(X_{n,k}\right)\ln\frac{E(X_{n,k})}{E(X)}. \end{array}$$

Now using $X_{n,k}\overset{st}{\leq }X$ in above inequality, we get

$$\begin{array}{cc} H({\bar{F}}_{n,k}) & \leq -\int_{0}^{\infty }{\bar{F}}_{n,k}\left(x\right)\ln\bar{F}\left(x\right)\;dx-E\left(X_{n,k}\right)\ln\frac{E(X_{n,k})}{E(X)}\\ \; & \leq -\int_{0}^{\infty }\bar{F}\left(x\right)\ln\bar{F}\left(x\right)\;dx-E\left(X_{n,k}\right)\ln\frac{E(X_{n,k})}{E(X)}\\ \; & =H\left(\bar{F}\right)-E\left(X_{n,k}\right)\ln\frac{E(X_{n,k})}{E(X)}. \end{array}$$

This proves the result. □

**Proposition** **2.** *Let X>0 be associated with the density function f(x) and cumulative distribution function F(x). If $X_{n,k}\overset{st}{\leq }X,$ then,*

(20) $$H\left({\bar{F}}_{n,k},\bar{F}\right)\leq Ce^{H(f_{n,k},f)}.$$

*Here $H\left(f_{n,k},f\right)=-\int_{0}^{\infty }f_{n,k}\left(x\right)\ln f\left(x\right)dx$ denotes the measure of inaccuracy between the $n^{th}$ k-record value and the parent distribution (see Goel et al. [22]).*

**Proof**. Consider,

$$\begin{array}{cc} \int_{0}^{\infty }f_{n,k}\left(x\right)\ln\frac{f(x)}{{\bar{F}}_{n,k}\left(x\right)\ln\bar{F}\left(x\right)}dx & \geq \ln\frac{1}{\int_{0}^{\infty }{\bar{F}}_{n,k}\left(x\right)\ln\bar{F}\left(x\right)dx}\\ \; & =\ln\frac{1}{-H({\bar{F}}_{n,k},\bar{F})}\;. \end{array}$$

The inequality above results from the log-sum inequality [23]. Continuing, we get

$$\begin{array}{cc} \int_{0}^{\infty }f_{n,k}\left(x\right)\ln f\left(x\right)dx-\int_{0}^{\infty }f_{n,k}\left(x\right)\ln\left({\bar{F}}_{n,k}\left(x\right)\ln\bar{F}\left(x\right)\right)dx & \geq -\ln\left(-H({\bar{F}}_{n,k},\bar{F})\right) \end{array}$$

or

$$H\left(f_{n,k},f\right)+\int_{0}^{\infty }f_{n,k}\left(x\right)\ln\left({\bar{F}}_{n,k}\left(x\right)\ln\bar{F}\left(x\right)\right)dx\leq \ln\left(-H\left({\bar{F}}_{n,k},\bar{F}\right)\right)$$

Using $X_{n,k}\overset{st}{\leq }X,$ we get

(21) $$H\left(f_{n,k},f\right)+\int_{0}^{\infty }f_{n,k}\left(x\right)\ln\left({\bar{F}}_{n,k}\left(x\right)\ln{\bar{F}}_{n,k}\left(x\right)\right)dx\leq \ln\left(-H\left({\bar{F}}_{n,k},\bar{F}\right)\right)$$

Using the substitution ${\bar{F}}_{n,k}\left(x\right)=u,$ we get

$$\int_{0}^{\infty }f_{n,k}\left(x\right)\ln\left({\bar{F}}_{n,k}\left(x\right)\ln{\bar{F}}_{n,k}\left(x\right)\right)dx=\int_{0}^{1}\ln\left(u\ln u\right)du=k.\;\;\left(say\right)$$

Therefore, by inserting this value into (21), we get

$$H\left(f_{n,k},f\right)+k\leq \ln\left(-H\left({\bar{F}}_{n,k},\bar{F}\right)\right)$$

or

$$H\left({\bar{F}}_{n,k},\bar{F}\right)\leq C\;e^{H(f_{n,k},f)},$$

where C=expk is a constant. □

**Proposition** **3.** *Let X be a non-negative random variable obeying the Weibull distribution; then*

(22) $$H\left({\bar{F}}_{n,k}\right)\leq \frac{E_{f_{n,k}}\left(X^{\beta +1}\right)\Gamma^{\beta }\left(1+\frac{1}{\beta }\right)}{\left(\beta +1\right)E^{\beta }\left(X_{n,k}\right)}\;.$$

**Proof**. Let *X* be a random variable obeying the Weibull distribution [24] and reliability function $\bar{F}\left(x\right)={e^{-(\lambda x)}}^{\beta }$.

From (18),

$$H\left({\bar{F}}_{n,k}\right)\leq -\int_{0}^{\infty }{\bar{F}}_{n,k}\left(x\right)\ln\bar{F}\left(x\right)\;dx-E\left(X_{n,k}\right)\ln\frac{E(X_{n,k})}{E(X)}\;.$$

The Weibull distribution becomes

$$\begin{array}{cc} -H({\bar{F}}_{n,k}) & \geq E\left(X_{n,k}\right)\ln\frac{E(X_{n,k})}{E(X)}-\int_{0}^{\infty }{\bar{F}}_{n,k}\left(x\right){\left(\lambda x\right)}^{\beta }dx\\ \; & =E\left(X_{n,k}\right)\ln\frac{E(X_{n,k})}{E(X)}-\frac{\lambda^{\beta }}{\beta +1}E_{f_{n,k}}\left(X^{\beta +1}\right). \end{array}$$

Let $E\left(X\right)=\mu =\int_{0}^{\infty }\bar{F}\left(x\right)dx=\frac{\Gamma (\frac{1}{\beta }+1)}{\lambda }$. Hence,

(23) $$-H\left({\bar{F}}_{n,k}\right)\geq E\left(X_{n,k}\right)\ln\frac{E(X_{n,k})}{\mu }-\frac{E_{f_{n,k}}\left(X^{\beta +1}\right)\Gamma^{\beta }\left(1+\frac{1}{\beta }\right)}{\left(\beta +1\right)\mu^{\beta }}\;.$$

The right-hand side of the above equation is maximized for a fixed *β* at

$$\mu_{\beta }={\left(\frac{\beta E_{f_{n,k}}\left(X^{\beta +1}\right)\Gamma^{\beta }\left(1+\frac{1}{\beta }\right)}{\left(\beta +1\right)E\left(X_{n,k}\right)}\right)}^{\frac{1}{\beta }}\;.$$

By inserting this result into (23), we get

$$\begin{array}{cc} -H({\bar{F}}_{n,k}) & \geq E\left(X_{n,k}\right)\ln\frac{E(X_{n,k})}{\mu_{\beta }}-\frac{E_{f_{n,k}}\left(X^{\beta +1}\right)\Gamma^{\beta }\left(1+\frac{1}{\beta }\right)}{\left(\beta +1\right)\mu_{\beta }^{\beta }}\\ \; & =-\frac{E(X_{n,k})}{\beta }\ln\left(\frac{\beta E_{f_{n,k}}\left(X^{\beta +1}\right)\Gamma^{\beta }\left(1+\frac{1}{\beta }\right)}{\left(\beta +1\right)E^{\beta +1}\left(X_{n,k}\right)}\right)-\frac{E(X_{n,k})}{\beta }\;. \end{array}$$

Now we know that −lnx≥1−x; using this, we get

$$\begin{array}{cc} -H({\bar{F}}_{n,k}) & \geq \frac{E(X_{n,k})}{\beta }\left(1-\frac{\beta E_{f_{n,k}}\left(X^{\beta +1}\right)\Gamma^{\beta }\left(1+\frac{1}{\beta }\right)}{\left(\beta +1\right)E^{\beta +1}\left(X_{n,k}\right)}\right)-\frac{E(X_{n,k})}{\beta }\\ \; & =\frac{-E_{f_{n,k}}\left(X^{\beta +1}\right)\Gamma^{\beta }\left(1+\frac{1}{\beta }\right)}{\left(\beta +1\right)E^{\beta }\left(X_{n,k}\right)}\;.\\ or\;\;\;H({\bar{F}}_{n,k}) & \leq \frac{E_{f_{n,k}}\left(X^{\beta +1}\right)\Gamma^{\beta }\left(1+\frac{1}{\beta }\right)}{\left(\beta +1\right)E^{\beta }\left(X_{n,k}\right)}\;. \end{array}$$

This completes the proof. □

**Remark** **3.** *If we set β=1 and n=k=1 in the previous result, it reduces to*

(24) $$H\left(\bar{F}\right)\leq \frac{E(X^{2})}{2E(X)}\;,$$

*a bound obtained by Rao et al. [3].*

**Proposition** **4.** *Suppose that the non-negative random variable X has a decreasing hazard rate; then*

(25) $$H\left({\bar{F}}_{n+1,k},\bar{F}\right)\leq H\left({\bar{F}}_{n,k},\bar{F}\right)$$

**Proof**. Consider two pdfs of consecutive record values $f_{n,k}\left(x\right)$ and $f_{n+1,k}\left(x\right)$. Using (5), we get

(26) $$\begin{array}{c} \frac{f_{n,k}\left(x\right)}{f_{n+1,k}\left(x\right)}=-\frac{n}{k\ln\bar{F}\left(x\right)}, \end{array}$$

which is a decreasing function in *x*. This implies that $X_{n,k}\overset{lr}{\leq }X_{n+1,k}$. Therefore $X_{n,k}\overset{st}{\leq }X_{n+1,k}$; that is ${\bar{F}}_{n,k}\left(x\right)\leq {\bar{F}}_{n+1,k}\left(x\right)$. (For more details one can refer to Shaked and Shantikumar [25].) Therefore for all increasing functions *ψ*, this is equivalent to $E\left(\psi \left(X_{n,k}\right)\right)\leq E\left(\psi \left(X_{n+1,k}\right)\right),$ provided these expectations exist.

Now if *X* has a decreasing hazard rate $\lambda_{F}\left(x\right)$, then $\frac{1}{\lambda_{F}\left(x\right)}$ is an increasing function. Therefore by the above,

$$E\left(\frac{1}{\lambda_{F}\left(X_{n,k}\right)}\right)\leq E\left(\frac{1}{\lambda_{F}\left(X_{n+1,k}\right)}\right)$$

From (9), we can see that

(27) $$H\left({\bar{F}}_{n,k},\bar{F}\right)\leq H\left({\bar{F}}_{n+1,k},\bar{F}\right)\;.$$

Here $\lambda_{F}\left(X_{n,k}\right)$ and $\lambda_{F}\left(X_{n+1,k}\right)$ denote the hazard rates corresponding to $X_{n,k}$ and $X_{n+1,k}$, respectively. This completes the proof. □

## 5. Cumulative Inaccuracy for Some Specific Distributions

In this section, we give a lemma providing a simplified expression for finding the cumulative inaccuracy measure for various distributions, and then we give some examples based on it.

The symbolic expressions and closed-form derivations of the inaccuracy measures $H({\bar{F}}_{n,k},\bar{F})$, including equations from Equation (29) to Equation (32), were performed using the Maple Computer Algebra System (Maplesoft, Version 2025.1). Maple’s symbolic integration, summation, and simplification capabilities were utilized to cross-check and symbolically collapse the infinite sums into closed-form expressions and checked via numerical substitution. The procedures closely follow the recommendations documented in the *Maple Programming Guide* [26].

**Lemma** **1.** *Consider a random variable X with distribution function $\bar{F}\left(x\right),$ where $F^{-1}\left(x\right)$ exists; then the cumulative inaccuracy measure between k-record values and the parent distribution is given as*

(28) $$H\left({\bar{F}}_{n,k},\bar{F}\right)=\sum_{j=0}^{n-1}\frac{k^{j}}{j!}\int_{0}^{\infty }\frac{u^{j+1}e^{-u(k+1)}}{f(F^{-1}\left(1-e^{-u}\right))}du\;.$$

**Proof**. From (8)

$$H\left({\bar{F}}_{n,k},\bar{F}\right)={\left(\bar{F}\left(x\right)\right)}^{k}\sum_{j=0}^{n-1}\int_{0}^{\infty }\frac{k^{j}}{j!}{\left(-\ln\bar{F}\left(x\right)\right)}^{j+1}dx\;.$$

By inserting $-\ln\bar{F}\left(x\right)=u$ into the previous equation, we get

$$\begin{array}{cc} H({\bar{F}}_{n,k},\bar{F}) & =e^{-ku}\sum_{j=0}^{n-1}\int_{0}^{\infty }\frac{k^{j}u^{j+1}e^{-u}}{j!f(F^{-1}\left(1-e^{-u}\right))}du\\ \; & =\sum_{j=0}^{n-1}\frac{k^{j}}{j!}\int_{0}^{\infty }\frac{u^{j+1}e^{-u(k+1)}}{f(F^{-1}\left(1-e^{-u}\right))}du\;. \end{array}$$

□

**Example** **1.** *Consider the finite range distribution with pdf $f\left(x\right)=\frac{a}{b}{\left(1-\frac{x}{b}\right)}^{a-1},\;\;a>1,\;\;0\leq x\leq b$ and distribution function $F\left(x\right)={\left(1-\frac{x}{b}\right)}^{a}.$*

*Then $\;\;\;F^{-1}\left(1-e^{-u}\right)=b\left(1-e^{-\frac{u}{a}}\right)$ and this gives $\;\;f\left(F^{-1}\left(1-e^{-u}\right)\right)=\frac{a}{b}e^{-\frac{u(a-1)}{a}}$. Therefore using (28), we get*

$$\begin{array}{cc} H({\bar{F}}_{n,k},\bar{F}) & =\sum_{j=0}^{n-1}\frac{k^{j}}{j!}\int_{0}^{\infty }\frac{u^{j+1}e^{-u(k+1)}}{f(F^{-1}\left(1-e^{-u}\right))}du\\ \; & =\sum_{j=0}^{n-1}\frac{k^{j}b}{j!a}\int_{0}^{\infty }\frac{u^{j+1}e^{-u(k+1)}}{e^{-\frac{u(a-1)}{a}}}du=\sum_{j=0}^{n-1}\frac{k^{j}b}{j!a}\int_{0}^{\infty }u^{j+1}e^{-u(k+\frac{1}{a})}du\;. \end{array}$$

*Now using the substitution $u(k+\frac{1}{a})=t$, we have*

(29) $$\begin{array}{cc} H({\bar{F}}_{n,k},\bar{F}) & =\sum_{j=0}^{n-1}\frac{k^{j}b}{j!a{\left(k+\frac{1}{a}\right)}^{j+2}}\int_{0}^{\infty }t^{j+1}e^{-t}dt\\ \; & =\frac{b}{a}\sum_{j=0}^{n-1}\frac{k^{j}\left(j+1\right)}{{\left(k+\frac{1}{a}\right)}^{j+2}}\\ \; & =\frac{b\left[-\frac{\left(ka+n+1\right){\left(\frac{ka}{ka+1}\right)}^{n}a^{2}}{ka+1}+a^{2}\right]}{a}\\ \; & =-\frac{\left(\left(ka+n+1\right){\left(\frac{ka}{ka+1}\right)}^{n}-ka-1\right)ba}{ka+1} \end{array}$$

*Here $\int_{0}^{\infty }t^{j+1}e^{-t}dt=\Gamma \left(j+2\right)=\left(j+1\right)!$*

*In particular if n=2andk=1, then we have an inaccuracy measure between the second record value and the parent distribution as follows:*

(30) $$H\left({\bar{F}}_{2,1},\bar{F}\right)=\frac{b}{a{\left(1+\frac{1}{a}\right)}^{2}}\left(\sum_{j=1}^{3}\frac{j}{{\left(1+\frac{1}{a}\right)}^{j-1}}\right)=\frac{ba\;(6a^{2}+4a+1)}{{\left(a+1\right)}^{4}}\;.$$

**Example** **2.** *For a uniform distribution, if we put a=1 in (29), we get an inaccuracy measure corresponding to a uniform distribution as follows:*

$$H\left({\bar{F}}_{n,k},\bar{F}\right)=b\sum_{j=0}^{n-1}\frac{k^{j}\left(j+1\right)}{{\left(k+1\right)}^{j+2}}=b\left(1-\frac{\left(k+n+1\right){\left(\frac{k}{k+1}\right)}^{n}}{k+1}\right)\;.$$

**Example** **3.** *If X is a random variable with a Weibull distribution with a pdf of $f\left(x\right)=\alpha \beta x^{\beta -1}e^{-\alpha x^{\beta }}$, forx>0,α>0,β>0 and survival function $\bar{F}\left(x\right)=1-e^{-\alpha x^{\beta }}$, this gives $F^{-1}\left(1-e^{-u}\right)={\left(\frac{u}{\alpha }\right)}^{\frac{1}{\beta }}$. Therefore putting these values in (28), the inaccuracy measure will come out as*

(31) $$\begin{array}{cc} H({\bar{F}}_{n,k},\bar{F}) & =\sum_{j=0}^{n-1}\frac{k^{j}\Gamma \left(j+1+\frac{1}{\beta }\right)}{j!{\left(\alpha \beta^{\beta }\right)}^{\frac{1}{\beta }}k^{j+1+\frac{1}{\beta }}}\;.\\ \; & =\frac{n\beta k^{n}\Gamma \left(\frac{n\beta +\beta +1}{\beta }\right)}{\left(\beta +1\right)n!{\left(\alpha \beta^{\beta }\right)}^{\frac{1}{\beta }}k^{\frac{n\beta +\beta +1}{\beta }}}\\ \; & =\frac{\beta k^{\frac{-\beta -1}{\beta }}{\left(\alpha \beta^{\beta }\right)}^{-\frac{1}{\beta }})\Gamma \left(\frac{n\beta +\beta +1}{\beta }\right)}{(\beta +1)(n-1)!}\;. \end{array}$$

**Example** **4.** *If X is an exponentially distributed random variable, then by putting β=1 in (31), we get an inaccuracy measure corresponding to an exponential distribution as follows:*

(32) $$H\left({\bar{F}}_{n,k},\bar{F}\right)=\sum_{j=0}^{n-1}\frac{\left(j+1\right)}{k^{2}\alpha }=\frac{n(n+1)}{2k^{2}\alpha }\;.$$

**Remark** **4.** *If we put k=1, then $H({\bar{F}}_{n,k},\bar{F})$ becomes $H({\bar{F}}_{n},\bar{F})$, which represents the cumulative residual inaccuracy measure between the $n^{th}$ record value and the parent distribution, and if n=1, k=1, this represents the cumulative residual entropy given by Rao et al. [3].*

## 6. Application to Extremal Quantum Uncertainty

The framework introduced for cumulative residual inaccuracy (CRI) under *k*-record statistics admits a direct extension to quantify informational divergence in extremal quantum phenomena. Quantum information processing systems frequently exhibit rare, high-impact deviations such as maximum gate errors, decoherence spikes, or syndrome bursts during error-corrected qubit operations [27,28,29]. Such deviations, structurally analogous to *k*-record values, possess disproportionate informational weight within the noise ensemble [30].

Let ${\bar{F}}_{n,k}\left(x\right)$ denote the survival function of the $k^{th}$ record in the empirical quantum error distribution and $\bar{F}\left(x\right)$ represent the reference survival function under the baseline decoherence model. The residual inaccuracy functional,

(33) $$H\left({\bar{F}}_{n,k},F\right)=-\int_{0}^{\infty }{\bar{F}}_{n,k}\left(x\right)\ln F\left(x\right)dx\;,$$

acts as a scalar quantifier of discord between the statistical extremality observed experimentally and the theoretically predicted uncertainty field. The value of $H(F_{n,k},F)$ increases as the observed tail deviates from the expected exponential or sub-exponential structure characteristic of nominal quantum noise processes [31,32].

In practice, the empirical parameters constituting $F_{n,k}$ may be derived from extremal quantum error syndromes aggregated across multiple correction cycles or tomography datasets [33]. The reference model F(x), frequently exponential or Weibull-distributed, aligns with conventional assumptions for decoherence and relaxation dynamics in open quantum systems [34,35]. Evaluation of (33) thus measures the incremental uncertainty generated by the discrepancies between the empirical and modeled error extremality.

For a simple exponential case, where the empirical and theoretical densities are, respectively, defined as $f\left(x\right)=\lambda e^{-\lambda x}$ and $g\left(x\right)=\lambda_{0}e^{-\lambda_{0}x}$, the CRI reduces to

(34) $$H\left(f,g\right)=-\log\lambda_{0}+\frac{\lambda_{0}}{\lambda },$$

illustrating that greater extremal variability ($\lambda <\lambda_{0}$) produces heightened uncertainty, indicating deviation from the designed fault-tolerant threshold [27,28].

The established formulation reframes the record-based uncertainty as an *entropic performance indicator* for quantum devices. Unlike mean-based entropies that track average uncertainty, the CRI integrates residual deviation structures, capturing both the persistence and asymmetry of extremal quantum events. This transition from expectation-based to tail-based informational analysis enables refined assessment of quantum reliability, measurement-chain stability, and the adequacy of noise models in practical implementations [36,37,38].

Thus, the cumulative residual inaccuracy measure derived here establishes a pragmatic statistical–entropic bridge between *record value information theory* and *quantum uncertainty quantification* [37,39]. It provides a continuous, interpretable diagnostic of noise asymmetry and extremal behavior in quantum devices—an attractive analytical tool for performance characterization, fault-tolerant design, and risk-aware quantum control.

### Relation to Entropic Uncertainty and the Logarithmic Schrödinger Equation

The CRI measure complements the broader framework of entropic uncertainty relations (EURs) in quantum mechanics, which quantify intrinsic limits on the joint knowledge of non-commuting observables through entropy sums or bounds [40,41,42]. The EUR or Hirschman uncertainty is defined as the sum of the temporal and spectral Shannon entropies. It turns out that Heisenberg’s uncertainty principle can be expressed as a lower bound on the sum of these entropies [43,44]. A related and insightful formulation arises from the *logarithmic Schrödinger equation* [45], which introduces a nonlinear quantum evolution incorporating the Shannon entropy functional into the dynamics. This equation underpins the Everett–Hirschman entropic uncertainty relation [42,44,46,47], which bounds the sum of the entropies of a quantum state’s position and momentum probability distributions, providing a stronger uncertainty bound than classical variance-based principles. This has invaluable applications in quantum memory and quantum information [40,47,48]. Moreover, the logarithmic Schrödinger equation has applications in fundamental physics ranging from superfluids to modeling the potential of the Higg’s boson [49,50,51].

While traditional entropic uncertainty relations operate in the phase space domain, assessing the incompatibility of observables, the CRI extends uncertainty quantification to *residual tail distributions* manifested in record statistics from sequences of quantum measurements or error syndromes. This links the entropic cost of observed extremal deviations to fundamental quantum limits framed by logarithmic nonlinearities in wavefunction evolution, thereby establishing a bridge from entropic uncertainty at the foundational level to operational uncertainty in quantum noise and error landscapes.

Integrating these perspectives enriches the interpretation of the CRI as an entropy-informed measure capturing layered quantum uncertainty from intrinsic measurement indeterminacy characterized by the Everett–Hirschman framework to emergent deviations evidenced in the cumulative residual inaccuracy of extremal events. This multiscale entropic viewpoint informs both the physical and statistical analysis of next-generation quantum information devices.

## 7. Conclusions

Record values and *k*-record values frequently arise in numerous practical scenarios. Examples include athletic competitions [52,53], hydrology [54,55], and weather forecasts [56]. Furthermore, Kerridge’s measure of inaccuracy [2] serves as a valuable tool for assessing the discrepancy between two distributions [2,57]. Therefore, in this communication, we present the cumulative residual inaccuracy between the distributions of the *k*-record values and the parent random variable. Furthermore, we derive several properties of this measure, including stochastic ordering. To reduce the computational effort required to determine the inaccuracy for various distributions, we offer a simplified expression for the proposed inaccuracy measure and demonstrate its application to several standard distributions.

## Data Availability

Data is contained within the article.

## Abbreviations

The following abbreviations are used in this manuscript:

CRE	Cumulative Residual Entropy
CRI	Cumulative Residual Innacuracy
DCI	Dynamic Cumulative Innacuracy
pdf	Probability Distribution Function
